# Native American Ancestry Is Associated with NAT2 Haplotype and Inferred Metabolizer Phenotype Variation in Latin American Populations

**DOI:** 10.3390/ijms27219884

**Published:** 2026-11-04

**Authors:** Mónica Acuña, Sergio Flores Carrasco

**Affiliations:** 1 Nucleo Interdisciplinario de Biologia y Genetica, Instituto de Ciencias Biomedicas (ICBM), Facultad de Medicina, Universidad de Chile, Independencia, Santiago 8380000, Chile; monica.acuna@uchile.cl; 2 Centro de Investigacion en Medicina de Altura, Vicerrectoria de Investigacion e Innovacion, Universidad Arturo Prat, Santiago 8320000, Chile

**Keywords:** NAT2, pharmacogenetics, ancestry, Latin America, inferred metabolizer phenotype, population genetics, precision medicine

## Abstract

The N-acetyltransferase 2 (NAT2) gene contributes to drug metabolism, yet its pharmacogenetic variation in admixed populations remains incompletely characterized. We evaluated associations of estimated Native American genetic ancestry with NAT2 haplotypes defined by seven single-nucleotide polymorphisms (SNPs) and inferred metabolizer phenotypes in four Admixed American (AMR) populations from the 1000 Genomes Project (n = 347). The complete Phase 3 dataset (n = 2504) provided comparative context. Haplotype distributions differed among AMR populations (χ^2^ = 78.61; Monte Carlo *p* = 1.0 × 10^−5^). After adjustment for sampling population, each 10-percentage-point increase in Native American ancestry was associated with lower odds of an inferred poor metabolizer phenotype (odds ratio [OR] = 0.78; 95% confidence interval [CI], 0.66–0.93; *p* = 0.006). NAT2*4.001-compatible and NAT2*7.002-compatible haplotypes were more frequent with higher Native American ancestry. The inferred poor metabolizer proportion was 57.5% in the lowest and 26.4% in the highest ancestry quartile. Excluding four individuals with indeterminate expanded coding-variant assignments yielded a similar association (n = 343; OR = 0.80; 95% CI, 0.67–0.95; *p* = 0.0097). These observational associations support further genotype-based investigation but do not establish causality or replace individual NAT2 genotyping.

## 1. Introduction

The N-acetyltransferase 2 (NAT2) gene encodes a polymorphic phase II enzyme involved in the metabolism of aromatic amines, hydrazines, and therapeutic drugs [1,2,3]. Combinations of coding variants give rise to NAT2 haplotypes with different functional activities. We use the terms inferred rapid, intermediate, and poor metabolizer, corresponding to the historical fast, intermediate, and slow acetylator categories, respectively [4,5]. These differences are pharmacologically relevant, particularly for drug-induced toxicity, but genotype-based phenotypes remain predictions rather than direct measurements of enzyme activity.

NAT2 haplotype frequencies differ markedly across geographical populations, reflecting demographic history, admixture, and potentially selection [6,7,8,9,10,11]. Previous studies have characterized NAT2 diversity in Indigenous American populations [8]. Updated nomenclature established by the Pharmacogene Variation Consortium (PharmVar) [12] and functional assignments provided by the Clinical Pharmacogenetics Implementation Consortium (CPIC) [13] offer a more consistent framework for interpreting common NAT2 haplotypes. Accordingly, population comparisons are informative, but haplotypes reconstructed using restricted single-nucleotide polymorphism (SNP) panels should be described as compatible with specific alleles rather than definitive molecular assignments.

Latin America is characterized by heterogeneous contributions from Indigenous American, European, African, and, to a lesser extent, other ancestral sources [14,15]. Population-genomic investigations reveal substantial variation both among and within sampling groups [16,17]. Recent work involving Andean and Amazonian groups has emphasized the clinical relevance of underrepresented Indigenous genomic diversity [18], while data from the Mexican Biobank demonstrate regional differences in medically relevant variation associated with demographic history and genetic ancestry [19]. Consequently, broad population labels alone may conceal pharmacogenetic heterogeneity [20,21].

Although NAT2 diversity has been studied across continents and in individual American populations [8,9], associations between individual-level Native American genetic ancestry and both NAT2 haplotypes and inferred metabolizer categories within admixed sampling groups remain incompletely characterized. Global ancestry proportions describe population history and should not be interpreted as causal determinants of NAT2 function or substitutes for genotyping [22,23]. Nevertheless, they offer a useful framework for investigating population-level associations in admixed groups.

The clinical relevance of NAT2 includes susceptibility to adverse reactions during treatment with drugs such as isoniazid, for which slow acetylation has been associated with hepatotoxicity [24,25]. The CPIC guideline for hydralazine also provides prescribing recommendations based on NAT2 genotype-predicted metabolizer status [5]. These applications highlight the importance of accurately characterizing NAT2 variation across diverse populations. Differences in predicted metabolizer distributions can inform future population pharmacogenomic research, although clinical outcomes were not measured here and individual treatment recommendations cannot be derived from ancestry proportions alone.

Our primary objective was to evaluate the association between individual Native American genetic ancestry and NAT2 seven-SNP haplotypes and inferred metabolizer phenotypes in the four Admixed American (AMR) populations of the 1000 Genomes Project: Colombians from Medellín, Colombia (CLM); Mexican Ancestry from Los Angeles, USA (MXL); Peruvians from Lima, Peru (PEL); and Puerto Ricans from Puerto Rico (PUR). We reconstructed seven-SNP haplotypes from previously phased genotype data, compared their frequencies among the four populations, and evaluated ancestry associations using population-adjusted models and comparisons between extreme ancestry quartiles. Comparisons with the other 1000 Genomes macro-populations and linkage disequilibrium analyses were used as contextual analyses rather than separate primary objectives.

## 2. Results

### 2.1. Differentiation Among the Four 1000 Genomes Admixed American Populations

Within AMR, seven-SNP NAT2 haplotype distributions differed significantly among CLM, MXL, PEL, and PUR (χ^2^ = 78.61, df = 18; Monte Carlo *p* = 1.0 × 10^−5^ based on 100,000 replicates), indicating substantial heterogeneity across the four sampled populations (Table 1).

PEL showed the highest frequencies of NAT2*4.001-compatible (46.5%) and NAT2*7.002-compatible (17.6%), whereas NAT2*6.002-compatible was more frequent in CLM (23.9%) and PUR (20.7%) than in PEL (8.2%). NAT2*1.001-compatible was particularly frequent in MXL (7.0%). Haplotype-specific heterogeneity remained significant after Benjamini–Hochberg false-discovery rate (FDR) correction for these four haplotypes. In contrast, NAT2*5.002-compatible, the most frequent haplotype in AMR (33.9%), showed no statistically significant differentiation among the four populations (Table 1).

Pairwise comparisons further characterized these differences, highlighting the distinctive PEL profile for NAT2*4.001-compatible, NAT2*6.002-compatible, and NAT2*7.002-compatible, as well as the higher NAT2*1.001-compatible frequency in MXL (Figure 1). Together, these results demonstrate that NAT2 haplotype distributions vary among the four AMR sampling groups rather than following a uniform regional pattern.

### 2.2. Association Between Native American Ancestry and NAT2 Variation

Within AMR individuals, higher Native American genetic ancestry was associated with lower odds of the inferred poor metabolizer phenotype. In an unadjusted logistic regression model, each 10-percentage-point increase in Native American ancestry was associated with lower odds of inferred poor metabolizer status (odds ratio [OR] = 0.83, 95% confidence interval [CI] 0.76–0.90, *p* = 1.1 × 10^−5^). This association remained significant after adjustment for AMR population (CLM, MXL, PEL, and PUR; OR = 0.78, 95% CI 0.66–0.93, *p* = 0.006). Including Native American ancestry significantly improved model fit compared with a population-only model (likelihood-ratio χ^2^ = 7.88, *p* = 0.005).

Haplotype-specific logistic regression analyses revealed associations between Native American ancestry and the carriage of several major NAT2 haplotypes after adjustment for AMR population (Figure 2). Higher Native American ancestry was positively associated with carriage of NAT2*7.002-compatible and NAT2*4.001-compatible haplotypes and inversely associated with NAT2*6.002-compatible and NAT2*5.002-compatible haplotypes. NAT2*1.001-compatible showed a positive point estimate, but its 95% CI included the null value. NAT2*5.001-compatible also showed an inverse association with Native American ancestry (OR = 0.43, 95% CI 0.23–0.78, FDR = 0.0089), although this estimate should be interpreted cautiously because only 12 individuals carried this haplotype.

### 2.3. Hardy–Weinberg Equilibrium

Multiallelic Hardy–Weinberg equilibrium (HWE) was evaluated separately in CLM, MXL, PEL, and PUR using reconstructed NAT2 diplotypes. In the primary probability-ordered conditional permutation analysis with 100,000 permutations, only PEL showed a statistically significant departure from HWE after Benjamini–Hochberg false-discovery rate (FDR) correction (permutation *p* = 0.0070, FDR = 0.028). No significant departures were detected in CLM (*p* = 0.142, FDR = 0.190), MXL (*p* = 0.278, FDR = 0.278), or PUR (*p* = 0.076, FDR = 0.152) (Table 2).

A complementary parametric Monte Carlo analysis based on Pearson’s chi-square statistic identified significant departures in both CLM (*p* = 0.00156, FDR = 0.00624) and PEL (*p* = 0.01789, FDR = 0.03578). In CLM, the parametric result was strongly influenced by a rare diplotype observed once despite a very low expected frequency. Given the sparse multiallelic genotype distributions and the sensitivity of Pearson’s chi-square statistic to rare genotype classes, the conditional permutation test was retained as the primary inferential procedure.

Descriptive diplotype diagnostics revealed observed and expected heterozygosities (Hₒ and Hₑ) of 0.638 and 0.719 in CLM, 0.750 and 0.748 in MXL, 0.682 and 0.676 in PEL, and 0.644 and 0.740 in PUR, respectively (Table 2). Therefore, the significant PEL departure identified by the primary conditional test did not correspond to a generalized heterozygote deficiency. In PEL (n = 85), homozygous NAT2*7.002-compatible diplotypes occurred 7 times versus 2.65 expected (8.2% versus 3.1%). NAT2*4.001/NAT2*5.002-compatible heterozygotes occurred 30 times versus 20.91 expected (35.3% versus 24.6%), whereas NAT2*5.002/NAT2*7.002-compatible diplotypes occurred 4 times versus 7.94 expected (4.7% versus 9.3%). These are descriptive genotype-level comparisons rather than individually tested departures.

### 2.4. Native American Ancestry Gradient Within Admixed American Individuals

Comparisons between individuals in the lowest (Q1) and highest (Q4) quartiles of Native American genetic ancestry revealed differences in the frequencies of several major NAT2 haplotypes. Individuals in Q4 showed higher frequencies of NAT2*4.001-compatible (45.9% vs. 25.9%) and NAT2*7.002-compatible (19.5% vs. 5.7%) haplotypes. Conversely, NAT2*6.002-compatible (6.9% vs. 23.0%) and NAT2*5.002-compatible (26.4% vs. 40.2%) were less frequent in Q4 than in Q1.

These patterns were consistent with the associations observed in the continuous-ancestry regression models. The four haplotype contrasts remained significant after Benjamini–Hochberg correction for multiple comparisons (Figure 3), supporting differentiation in NAT2 haplotype frequencies across the Native American ancestry gradient.

### 2.5. Association Between Native American Ancestry and Inferred NAT2 Metabolizer Phenotypes

Inferred NAT2 metabolizer phenotype distributions differed significantly between individuals in the lowest (Q1) and highest (Q4) quartiles of Native American genetic ancestry (χ^2^ = 17.23, df = 2, *p* = 1.81 × 10^−4^). In Q1, individuals classified as poor metabolizers represented the most frequent phenotype (57.5%), followed by intermediate (29.9%) and rapid metabolizers (12.6%). In contrast, intermediate metabolizers predominated in Q4 (52.9%), while the proportion of poor metabolizers was lower (26.4%) and that of rapid metabolizers was higher (20.7%). These findings indicate differences in inferred NAT2 metabolizer phenotype distributions across the Native American ancestry gradient (Table 3).

Multinomial logistic regression further supported these associations. After adjustment for AMR population (CLM, MXL, PEL, and PUR), each 10-percentage-point increase in Native American genetic ancestry was associated with higher odds of being classified as an intermediate rather than poor metabolizer (OR = 1.26, 95% CI 1.05–1.51, *p* = 0.0139) and as a rapid rather than poor metabolizer (OR = 1.34, 95% CI 1.03–1.73, *p* = 0.0275).

### 2.6. Regional Coding-Variant Assessment and Sensitivity Analysis

The regional 1000 Genomes Phase 3 variant call format (VCF) file contained the same 2504 individuals and 1603 variants. All 17,528 phased individual genotypes at the seven original SNPs were concordant with the original input. Forty-three variable coding sites were observed, including 36 outside the seven-SNP panel. The original panel yielded one individual with an indeterminate inferred phenotype globally and none among the 347 AMR participants. Expanded coding-core compatibility assessment, incorporating CPIC functional assignments, identified 56 individuals with indeterminate inferred phenotypes globally (2.24%), including four AMR participants (1.15%). These assignments involved 57 chromosome copies: 45 with uncertain function, four with unknown function, and eight with unresolved coding combinations. Expanded coding-core-compatible assignments within each original seven-SNP motif are summarized in Supplementary Table S1.

Among AMR participants, two individuals carried coding haplotypes compatible with the uncertain-function NAT2*43 or NAT2*44 alleles, whereas two carried unresolved coding combinations (Supplementary Table S2). Their original seven-SNP-based phenotypes were intermediate in three cases and rapid in one. After excluding these four individuals, 343 participants remained: 152 inferred poor, 143 intermediate, and 48 rapid metabolizers. No AMR participant with a determinate expanded functional assignment changed between resolved metabolizer categories. Three additional carriers of nonsynonymous variants retained decreased-function assignments under the expanded assessment.

The association between Native American genetic ancestry and inferred poor metabolizer status remained significant after adjustment for AMR population and exclusion of the four individuals with indeterminate expanded assignments (OR = 0.796 per 10-percentage-point increase in Native American ancestry, 95% CI 0.669–0.946, *p* = 0.0097; n = 343). Excluding all seven AMR carriers of additional nonsynonymous variants yielded a similar estimate (OR = 0.796, 95% CI 0.670–0.947, *p* = 0.0100; n = 340; Supplementary Table S3). Using the original ancestry-quartile thresholds, the proportion of inferred poor metabolizers remained at 57.5% in Q1 and was 26.7% in Q4 after exclusion of the four indeterminate individuals (three-category χ^2^ test, *p* = 0.000230). The original seven-SNP analyses remain the primary results, while these sensitivity analyses provide evidence of robustness to the additional coding variation observed in the regional dataset.

As an exploratory sensitivity analysis addressing the HWE departure detected in PEL, we refitted the population-adjusted logistic regression after excluding all 85 PEL participants. Among the 262 participants from CLM, MXL, and PUR, higher Native American ancestry remained associated with lower odds of inferred poor metabolizer status (OR per 10-percentage-point ancestry increase = 0.770; 95% CI, 0.626–0.947; *p* = 0.0132). This analysis indicates that the primary association was not driven exclusively by PEL; it does not establish that Hardy–Weinberg departures are irrelevant.

### 2.7. Contextual Comparisons Across 1000 Genomes Macro-Populations

For contextual comparison, NAT2 seven-SNP haplotype distributions were examined across the five 1000 Genomes macro-populations: African (AFR), Admixed American (AMR), East Asian (EAS), European (EUR), and South Asian (SAS). Haplotype distributions differed significantly across these groups (χ^2^ = 2072.71, df = 32, *p* < 0.001). All eight major haplotypes, defined by a global frequency of at least 1%, showed significant heterogeneity after Benjamini–Hochberg false-discovery rate correction across the 17 observed haplotype motifs (Table 4).

NAT2*4.001-compatible was most frequent in EAS (52.1%), NAT2*5.002-compatible in EUR (41.8%), and NAT2*6.002-compatible in SAS (35.7%). NAT2*7.002-compatible showed comparatively higher frequencies in EAS (18.0%) and AMR (11.2%) than in the other macro-populations. Pairwise comparisons between AMR and the other macro-populations revealed comparator-specific patterns of haplotype differentiation (Supplementary Figure S1, Supplementary File S2), providing population-genetic context for the primary within-AMR findings.

### 2.8. Linkage Disequilibrium Structure at the NAT2 Locus

Within AMR, the strongest pairwise linkage disequilibrium (LD) among the seven NAT2 SNPs was observed for rs1801280–rs1799929 (r^2^ = 0.93), followed by rs1801280–rs1208 (r^2^ = 0.90) and rs1799929–rs1208 (r^2^ = 0.84). Most other SNP pairs showed low-to-moderate LD. As this analysis provides complementary information on haplotype structure, the LD heatmaps are presented in Supplementary Figure S2 (Supplementary File S2).

LD patterns varied across the five 1000 Genomes macro-populations, with generally lower LD for several major SNP pairs in AFR and heterogeneous patterns across the remaining populations. These findings provide population-genetic context for the within-AMR analyses but do not constitute direct evidence of natural selection or local ancestry at the NAT2 locus.

## 3. Discussion

The primary finding of this study is that individual Native American genetic ancestry is associated with NAT2 seven-SNP haplotypes and inferred metabolizer categories within the four Admixed American populations represented in the 1000 Genomes Project. Higher Native American ancestry was associated with lower odds of an inferred poor metabolizer phenotype after adjustment for sampling population (OR = 0.78 per 10-percentage-point increase in ancestry; *p* = 0.006). This association builds on previous studies of geographical NAT2 differentiation by examining individual ancestry variation within admixed populations rather than relying exclusively on broad population labels [8,9,11].

NAT2 haplotype distributions differed among the four AMR populations. PEL showed higher frequencies of NAT2*4.001-compatible and NAT2*7.002-compatible haplotypes, whereas NAT2*6.002-compatible was more frequent in CLM and PUR, and NAT2*1.001-compatible was particularly enriched in MXL. These patterns are consistent with the heterogeneous demographic histories documented in Latin American populations [14,15,26]. Recent large-scale genomic studies of Mexico further illustrate the importance of individual ancestry variation and regional sampling, which may be obscured by broad national or continental classifications [17,19].

The consistency between continuous-ancestry regression models and comparisons of extreme ancestry quartiles supports the observed ancestry-associated variation in NAT2. The proportion of individuals classified as inferred poor metabolizers was substantially lower in Q4 (26.4%) than in Q1 (57.5%). Nevertheless, these associations are observational and should not be interpreted as evidence of a causal effect of genetic ancestry on NAT2 function. Differences in haplotype frequencies across the five 1000 Genomes macro-populations are consistent with previously described patterns of NAT2 population differentiation [6,9]. Together with the linkage disequilibrium profiles, these comparisons provide population-genetic context for the within-AMR findings but do not distinguish the demographic or selective processes underlying the observed variation [27,28].

The interpretation of the Native American ancestry component is consistent with the original 1000 Genomes Phase 3 study, which identified Native American admixture in populations from the Americas using unsupervised ancestry inference [29]. The population-level distribution observed in our ancestry estimates provides additional support for this interpretation. However, this methodological precedent does not constitute independent quantitative validation of the individual ancestry proportions estimated using our ancestry-informative marker panel. Global genetic ancestry reflects shared population history and genomic structure, and its association with NAT2 variation may partly reflect correlations among ancestral components and residual population structure despite adjustment for sampling population. Importantly, these findings neither establish local ancestry at the NAT2 locus nor demonstrate causality. Broader representation of Indigenous American populations, including Andean and Amazonian groups, will be important for further investigating these relationships [18].

The expanded regional coding-variant assessment provided additional support for the robustness of the primary association. The association persisted after conservative exclusion of four AMR participants with indeterminate functional assignments and after exclusion of all seven carriers of additional nonsynonymous variants. These findings indicate that the observed association is not substantially affected by the additional coding variation identified in the regional dataset. However, they do not establish definitive PharmVar suballele assignments or demonstrate the clinical validity of the seven-SNP panel.

Despite this robustness analysis, seven coding SNPs are insufficient for definitive resolution of all current NAT2 alleles, and the resulting acetylator categories remain inferred rather than clinically observed. Our data do not include measured acetylation activity, drug exposure, adverse events, or treatment outcomes. Clinical and functional validation will require broader sequencing and prospectively phenotyped cohorts; the present results are intended to guide population-level investigations, not treatment recommendations.

The departure from Hardy–Weinberg equilibrium observed in PEL using the primary conditional test should be interpreted cautiously. A complementary parametric analysis also identified a deviation in CLM, largely driven by a rare diplotype with a very low expected frequency. These differences highlight the sensitivity of multiallelic HWE testing to sparse genotype distributions. Population substructure, nonrandom mating, and technical factors affecting genotype or phasing accuracy remain possible explanations, and neither result alone establishes natural selection at NAT2.

The PEL genotype-level diagnostics suggest an alteration in the distribution of particular reconstructed NAT2 diplotypes rather than an overall excess of homozygosity, as Hₒ and Hₑ were closely similar. Sparse expected diplotype counts, population substructure, non-random mating, and uncertainties concerning genotype calling or phasing remain alternative explanations; the present data cannot distinguish them. An exploratory exclusion of PEL preserved the direction and significance of the ancestry association (OR = 0.770, *p* = 0.0132). Neither these results nor the HWE departure constitute evidence of selection or validate individual clinical prediction.

The pharmacological relevance of NAT2 is supported by its established association with susceptibility to anti-tuberculosis drug-induced hepatotoxicity [24,25] and by the CPIC guideline providing genotype-based prescribing recommendations for hydralazine [5]. Nevertheless, the population-level associations identified here do not justify individual treatment recommendations. Clinical interpretation requires individual NAT2 genotyping, sufficient variant coverage, and appropriate functional classification. Global ancestry estimates cannot substitute for individual genotype information or directly measured metabolic phenotypes. Similar concerns regarding pharmacogenetic variant coverage and population diversity have been discussed for other pharmacogenes, including DPYD [30]. Evidence from broader pharmacogenetic implementation studies [31,32] must also be distinguished from NAT2-specific evidence. In particular, the Preemptive Pharmacogenomic Testing for Preventing Adverse Drug Reactions (PREPARE) study did not include NAT2 in its 12-gene panel and therefore does not provide direct evidence of NAT2-specific clinical utility.

Several limitations should be considered. The 347 AMR participants represent four specific 1000 Genomes sampling groups rather than nationally representative populations or Latin America as a whole. The expanded coding-variant assessment relied on a regional variant-only VCF derived from Phase 3 sequencing data; the absence of a variant record does not establish sufficient coverage or exclude additional genetic variation. Rare variants, phasing accuracy, structural and regulatory variation, and complete NAT2 suballeles were not comprehensively validated, and metabolizer phenotypes were not measured experimentally. Furthermore, the ancestry estimates were obtained using an unsupervised approach and interpreted as broad ancestral components. Individual proportions were not independently benchmarked against an external Indigenous American reference panel, and local ancestry at the NAT2 locus was not evaluated. Sparse haplotype categories and modest within-population sample sizes impose additional limitations on statistical precision. Larger studies incorporating comprehensive NAT2 characterization, local ancestry analysis, diverse population sampling, and clinical endpoints will be needed to clarify the biological and pharmacological implications of these findings [16,22,33].

## 4. Materials and Methods

The primary analysis concerned 347 individuals from the four AMR populations represented in the 1000 Genomes Project (1KGP) Phase 3: Colombians from Medellín (CLM), individuals of Mexican ancestry from Los Angeles (MXL), Peruvians from Lima (PEL), and Puerto Ricans from Puerto Rico (PUR). Contextual haplotype and LD analyses included the entire phased dataset of 2504 individuals across five macro-populations: Africans (AFR), Admixed Americans (AMR), East Asians (EAS), Europeans (EUR), and South Asians (SAS).

### 4.1. NAT2 SNP Selection, Haplotype Reconstruction, and Functional Annotation

The NAT2 dataset comprised seven previously characterized SNPs: rs1801279, rs1041983, rs1801280, rs1799929, rs1799930, rs1208, and rs1799931. These correspond to the conventional NAT2 coding-sequence nucleotide positions 191, 282, 341, 481, 590, 803, and 857, respectively. Chromosome-specific seven-SNP haplotypes were reconstructed directly from the phased genotype data provided by the 1000 Genomes Project, without additional statistical phasing. This procedure yielded 5008 chromosome-level haplotypes across the 2504 individuals.

Observed seven-SNP motifs were mapped to historical NAT2 star-allele labels and PharmVar-compatible allele assignments. Because the seven-SNP panel does not capture all variants required to distinguish current PharmVar suballeles, these assignments were considered compatible with, rather than definitive for, specific alleles or suballeles [12]. Historical allele labels were retained to facilitate comparisons with earlier NAT2 literature. Motifs that could not be assigned unambiguously from the seven-SNP panel were classified as unresolved.

For the primary panel-based analysis, PharmVar-compatible allele families were provisionally assigned functional categories according to CPIC classifications [13]. Motifs compatible with the NAT2*1 and NAT2*4 families were classified as increased function, whereas those compatible with NAT2*5, NAT2*6, NAT2*7, NAT2*14, NAT2*16, NAT2*34, and NAT2*46 were classified as decreased function. Individual diplotypes were reconstructed by pairing the two chromosome-specific haplotypes carried by each individual.

Metabolizer phenotypes were inferred from the functional combination of the two assigned alleles. Individuals carrying two increased-function alleles were classified as rapid metabolizers, those carrying one increased-function and one decreased-function allele as intermediate metabolizers, and those carrying two decreased-function alleles as poor metabolizers. Individuals carrying an unresolved allele were classified as indeterminate and excluded from phenotype-based analyses.

These phenotype assignments represent genotype-based predictions derived from the seven-SNP panel rather than direct measurements of enzymatic activity. Variants not captured by the panel may refine allele assignments and, in some instances, modify the predicted functional category. Throughout the manuscript, metabolizer categories derived from these diplotypes are therefore referred to as inferred phenotypes unless otherwise specified. The primary analysis excluded one individual with an indeterminate assignment globally and none among the 347 AMR participants. The expanded regional coding-variant assessment described in Section 4.10 evaluated additional observed coding variation and applied conservative sensitivity exclusions without replacing the primary seven-SNP analyses.

### 4.2. Population Assignment and Global Ancestry Estimation

Population labels and individual genetic ancestry estimates were integrated with the NAT2 dataset using sample identifiers. The five 1000 Genomes macro-populations were AFR, AMR, EAS, EUR, and SAS, comprising 26 Phase 3 populations. The primary analyses focused on the four AMR populations: CLM, MXL, PEL, and PUR.

For the ancestry-based analyses, we used previously generated individual genetic ancestry estimates rather than performing new ancestry estimation. These estimates were based on a panel of 446 ancestry-informative markers (AIMs) originally developed and validated to characterize admixture in Latin American populations, particularly the major Native American, European, and African ancestral contributions [34]. Ancestry proportions had been estimated using STRUCTURE v2.3.4 [35] under an unsupervised admixture model with K = 5, employing 10,000 burn-in iterations followed by 100,000 iterations for parameter estimation, consistent with the methodological framework used in our previous studies [36,37].

During the earlier ancestry analyses, exploratory solutions up to K = 7 were examined. The K = 5 solution yielded an interpretable Native American-associated component, characterized by a broad population-level pattern of PEL > MXL > CLM > PUR. At K = 6 and K = 7, additional components were interpreted as primarily subdividing African-associated ancestry. These observations informed the previous selection of K = 5. However, the higher-K outputs were not retained, and the original model-selection assessment cannot be independently reproduced from the currently available files. Therefore, K = 5 is presented as the previously selected analytical solution rather than as a statistically demonstrated optimal number of ancestral components.

The five inferred components were labeled post hoc as European, East Asian, South Asian, Native American, and African based on their distribution across the 1000 Genomes populations. Although the original AIM panel was developed using African, European, and Native American reference populations [34], no external Indigenous American reference individuals were included to supervise the STRUCTURE estimates reused in the present study. The interpretation of the Native American-associated component was supported by its enrichment in PEL and the observed population-level distribution across the four AMR samples.

A methodological precedent is provided by the original 1000 Genomes Phase 3 analysis, which used unsupervised ADMIXTURE (K = 5–12) on 193,634 autosomal variants from 2504 individuals representing 26 populations and identified population structure consistent with Native American admixture in samples from the Americas [29]. This analysis is described in Section 9.7 (“Assessment of population structure using ADMIXTURE”) of the Supplementary Information accompanying that publication and illustrated in its Extended Data Figure 5. This precedent supports the population-level interpretation of the component but does not independently validate the individual ancestry proportions estimated using our AIM panel.

The merged dataset contained population labels and ancestry estimates for all 2504 individuals. Within AMR, Native American genetic ancestry was examined as a continuous variable and through comparisons between extreme ancestry quartiles. Individuals were ranked according to their estimated Native American ancestry proportions, and those belonging to the lowest (Q1) and highest (Q4) quartiles were compared as a complementary descriptive analysis.

### 4.3. Haplotype Frequency Estimation and Population Differentiation

Haplotype frequencies were calculated at the chromosome level for AFR, AMR, EAS, EUR, and SAS. Major haplotypes were defined as those with a global frequency of at least 1%, whereas rarer motifs, their chromosome counts, and expanded coding-core-compatible assignments, including unresolved combinations, are reported in Supplementary Table S1. Haplotype-specific heterogeneity across macro-populations was evaluated using Pearson chi-square tests comparing each of the 17 observed seven-SNP motifs against all remaining motifs across the five macro-populations. Benjamini–Hochberg false-discovery rate (FDR) correction [38] was applied across all 17 tests. Table 4 presents the eight motifs with global frequencies ≥1%. Given the sparse expected counts for some rare motifs, emphasis was placed on the more frequent haplotypes.

An omnibus haplotype-by-macro-population test was additionally performed. Haplotypes with a global frequency below 1% were grouped into an “Other” category to satisfy expected-count assumptions before applying the Pearson chi-square test. Post hoc comparisons were conducted between AMR and each of AFR, EAS, EUR, and SAS. For each of the 17 haplotypes and four population comparisons, Fisher’s exact test was applied to a 2 × 2 contingency table, yielding 68 comparisons. Effect direction and magnitude were summarized as the log_2_ ratio of AMR to comparator haplotype frequency. For graphical representation, a uniform 0.5 pseudocount was added to haplotype counts, with one added to each population denominator, whereas Fisher’s exact tests used unadjusted counts. Benjamini–Hochberg correction was applied across all 68 comparisons.

Within AMR, haplotype frequencies were estimated separately for CLM, MXL, PEL, and PUR. An omnibus heterogeneity test was performed after grouping haplotypes with AMR frequencies below 1% into an “Other” category. Because sparse expected counts remained after grouping, statistical significance was evaluated using a Pearson chi-square statistic with a Monte Carlo *p*-value based on 100,000 replicates. Haplotype-specific heterogeneity was additionally assessed using Pearson chi-square tests comparing each of the ten observed AMR motifs against all remaining motifs across the four populations. Benjamini–Hochberg correction was applied across these ten tests, as reported in Table 1. Results for low-frequency motifs were interpreted cautiously because of sparse expected counts.

All six pairwise population contrasts among CLM, MXL, PEL, and PUR were subsequently evaluated using Fisher’s exact tests for each of the ten observed AMR haplotype motifs, yielding 60 comparisons. Effect sizes were expressed as the log_2_ frequency ratio between the first and second population in each contrast, with a uniform 0.5 pseudocount added to both haplotype counts and one added to both population denominators for graphical representation only. Fisher’s exact tests used the original counts. A single Benjamini–Hochberg correction was applied across all 60 pairwise tests.

### 4.4. Association of Native American Ancestry with Inferred Metabolizer Phenotype

Ancestry-based analyses were restricted to the 347 AMR individuals with determinate inferred NAT2 metabolizer phenotypes. Native American genetic ancestry was modeled as a continuous variable, scaled in 10-percentage-point units to facilitate interpretation of effect estimates.

Binary logistic regression was used to evaluate the association between Native American ancestry and inferred poor metabolizer status, defined as poor versus intermediate or rapid metabolizer phenotypes combined. An unadjusted model was initially fitted, followed by a model including AMR population (CLM, MXL, PEL, and PUR) as a categorical covariate. A likelihood-ratio test compared the population-only model with the model additionally including Native American ancestry to assess its contribution to model fit.

An additional exploratory sensitivity regression excluded PEL, the only AMR group with a significant departure in the primary multiallelic HWE test. The remaining 262 participants were analyzed with the same binary outcome, Native American ancestry scaling (per 10 percentage points), and categorical adjustment for CLM, MXL, and PUR. This analysis was performed using the same phased NAT2 genotypes and individual ancestry data as the primary model.

Multinomial logistic regression was subsequently performed to examine associations across the three inferred metabolizer categories. Poor metabolizer status was used as the reference category, with intermediate and rapid metabolizer phenotypes modeled as alternative outcomes. Native American ancestry, scaled per 10-percentage-point increase, was included as the predictor of interest, with AMR population as a categorical covariate.

Odds ratios (ORs) and corresponding 95% confidence intervals (CIs) were obtained by exponentiating regression coefficients and their Wald confidence limits, respectively. Two-sided Wald *p*-values were calculated for the ancestry coefficients. The multinomial model estimates were interpreted as relative odds of intermediate or rapid metabolizer status compared with poor metabolizer status.

### 4.5. Haplotype-Specific Ancestry Associations

To characterize associations between Native American genetic ancestry and individual NAT2 haplotypes, separate binary logistic regression models were fitted for each of the six major AMR haplotypes, defined as those with a chromosome-level frequency of at least 1%. For each model, the dependent variable was haplotype carrier status, defined as the presence of at least one copy of the corresponding seven-SNP haplotype. Individuals without the haplotype served as the reference group.

Native American ancestry, scaled per 10-percentage-point increase, was included as the predictor of interest, with AMR population (CLM, MXL, PEL, and PUR) included as a categorical covariate. Adjusted odds ratios (ORs) and 95% confidence intervals (CIs) were obtained from logistic regression coefficients and their Wald standard errors. Two-sided Wald *p*-values were adjusted across the six haplotype-specific models using the Benjamini–Hochberg false-discovery rate procedure. These analyses were interpreted as associations between genetic ancestry and haplotype carriage rather than evidence of causal effects.

### 4.6. Extreme-Quartile Ancestry Analysis

As a complementary descriptive analysis, the 347 AMR individuals were stratified according to their estimated Native American genetic ancestry proportions. Individuals belonging to the lowest (Q1) and highest (Q4) ancestry quartiles were selected for comparison, using ancestry thresholds of ≤12.55% and ≥57.30%, respectively. Each quartile comprised 87 individuals, corresponding to 174 chromosome copies per group.

NAT2 haplotype frequencies were compared between Q1 and Q4 using Fisher’s exact tests applied to chromosome-level counts. Eight haplotype motifs were observed in at least one of the two extreme quartiles, and their *p*-values were adjusted using the Benjamini–Hochberg false-discovery rate procedure. Effect direction and magnitude were summarized as log_2_(Q1/Q4) haplotype frequency ratios. For graphical representation, a uniform 0.5 pseudocount was added to haplotype counts, with one added to each quartile denominator, whereas Fisher’s exact tests used the original counts.

The distributions of inferred NAT2 metabolizer phenotypes (poor, intermediate, and rapid) were compared between Q1 and Q4 using Pearson’s chi-square test of independence. These extreme-quartile comparisons were considered descriptive and complementary to the primary population-adjusted regression analyses.

### 4.7. Hardy–Weinberg Equilibrium and Diagnostic Analyses

Multiallelic Hardy–Weinberg equilibrium (HWE) was evaluated separately in CLM, MXL, PEL, and PUR using reconstructed NAT2 diplotypes. Because several multiallelic genotype classes had low expected counts, the primary analysis employed a probability-ordered conditional genotype-permutation procedure implemented in the accompanying R script, following the multiallelic exact-testing framework of Guo and Thompson [39].

For each population, the procedure conditioned on the observed haplotype allele counts and generated 100,000 random genotype pairings using fixed population-specific random seeds. Statistical significance was assessed by comparing the conditional probabilities of simulated genotype configurations with that of the observed configuration. Monte Carlo *p*-values were calculated using a correction for the finite number of simulations and adjusted across the four populations using the Benjamini–Hochberg false-discovery rate procedure. Observed and expected heterozygosity were also calculated.

As a complementary diagnostic analysis, a parametric Monte Carlo procedure was performed using Pearson’s chi-square statistic under fitted HWE genotype frequencies. For each population, 100,000 genotype samples were simulated, with allele frequencies re-estimated within each simulated dataset. This analysis was used to assess the sensitivity of HWE results to an alternative test statistic and simulation framework. Given the presence of sparse genotype classes, the probability-ordered conditional test was retained as the primary inferential procedure.

To characterize the source of the multiallelic HWE departure descriptively, population-specific diplotype counts were tabulated and compared with genotype frequencies expected under HWE from observed haplotype frequencies (pᵢ). For homozygous diplotypes, the expected count was n × pᵢ^2^; for heterozygous diplotypes, it was n × 2pᵢpⱼ. Observed heterozygosity (Hₒ) was the proportion of individuals carrying two distinct haplotypes; expected heterozygosity (Hₑ) was 1 − Σpᵢ^2^. Because several expected diplotype counts were small, individual genotype contrasts were described without additional significance tests.

### 4.8. Linkage Disequilibrium Analysis

Linkage disequilibrium (LD) was evaluated among the seven NAT2 SNPs included in the haplotype analysis (rs1801279, rs1041983, rs1801280, rs1799929, rs1799930, rs1208, and rs1799931). Using the previously phased genotype data, the two chromosome-specific allele copies from each individual were represented separately. For each pair of biallelic SNPs, LD was quantified as the squared Pearson correlation coefficient (r^2^) between allele indicators across chromosomes.

Pairwise LD estimates were calculated separately for the five 1000 Genomes macro-populations (AFR, AMR, EAS, EUR, and SAS) and for the complete dataset of 2504 individuals. SNP pairs involving a monomorphic marker within a population were assigned undefined r^2^ values rather than zero. This occurred for rs1801279 in EAS and SAS. The resulting pairwise r^2^ matrices were visualized as heatmaps to compare LD patterns across populations (Supplementary Figure S2, Supplementary File S2).

Particular attention was given to the AMR population to characterize the LD structure among the analyzed NAT2 variants. These analyses were considered descriptive and complementary to the primary ancestry-association analyses and were not intended to establish local ancestry or provide direct evidence of natural selection.

### 4.9. Computational Implementation and Reproducibility

The original NAT2 genotype extraction and preprocessing were performed using VCFtools v0.1.16 [40]. To document computational reproducibility, the primary analyses were consolidated into a single R script, successfully executed under R v4.6.1 using base R and the nnet package for multinomial logistic regression. The script processes the phased seven-SNP NAT2 variant call format (VCF) file and the corresponding individual ancestry and population data, reconstructs haplotypes and inferred metabolizer phenotypes, performs haplotype frequency comparisons across populations, fits binary and multinomial regression models, evaluates extreme ancestry quartiles, and calculates linkage disequilibrium.

Multiallelic Hardy–Weinberg equilibrium was evaluated using 100,000 probability-ordered conditional genotype permutations per AMR population, supplemented by 100,000 parametric Monte Carlo simulations per population. The omnibus haplotype comparison among the four AMR populations was evaluated using 100,000 Monte Carlo replicates. Fixed random seeds were used for the simulation procedures. The script includes automated numerical checks against selected principal manuscript results and records the computational environment through R session information. A completed execution reproduced all deterministic numerical checks implemented in the script, and the documented fixed-seed run yielded the primary HWE permutation estimates reported in Table 2.

Supplementary File S1 provides the consolidated R analysis script (R version 4.6.1), the phased seven-SNP NAT2 VCF, the corresponding individual ancestry and population data, execution instructions, and SHA256 checksums. The ancestry estimates were generated in the previous studies described in Section 4.2 and were reused as input data. Supplementary File S4 provides an independent Python implementation, using NumPy and SciPy, to reproduce the original population-adjusted association and the sensitivity analysis excluding the four AMR individuals with indeterminate expanded functional assignments. This implementation was independently verified using Python version 3.13.5, NumPy version 2.3.5, and SciPy version 1.17.0. Expanded coding-core compatibility and additional sensitivity results are reported in Supplementary File S3.

AI-assisted support during manuscript revision. ChatGPT (https://chatgpt.com, OpenAI, web interface; accessed September–October 2026) was used to assist with scientific-language editing, code refinement, computational consistency checks, and preparation of reproducibility documentation and Python-based regression sensitivity workflows. Statistical analyses were performed using R (version 4.6.1), and independent computational verification was conducted using Python (version 3.13.5), NumPy (version 2.3.5), and SciPy (version 1.17.0). The AI tool did not generate or modify the original 1000 Genomes genotype data or the previously estimated genetic ancestry proportions. The authors reviewed the computational procedures and their outputs and retain full responsibility for the methods, statistical results, interpretations, and final manuscript.

### 4.10. Regional Coding-Variant Assessment

The original seven-SNP dataset was compared with a regional 1000 Genomes Phase 3 variant call format (VCF) file (Genome Reference Consortium human build 37 [GRCh37], chromosome 8:18,235,000–18,275,000; v5b release) by matching sample identifiers and phased reference (REF) and alternate (ALT) allele sequences. Coding haplotypes were reconstructed across the NAT2 coding sequence (GRCh37, chromosome 8:18,257,514–18,258,386, positive strand). The resulting coding-variant profiles were compared with PharmVar NAT2 core allele definitions (version 6.2.29) and the CPIC NAT2 allele functionality reference table, both accessed on 14 October 2026.

Coding-core compatibility was assessed by comparing the observed variant combinations at PharmVar core-defining positions. Positions without recorded variants in the regional VCF were provisionally treated as reference alleles for compatibility matching, without assuming that their sequencing coverage was sufficient to exclude additional variation. Synonymous differences were not used to distinguish core alleles in the expanded assessment. Additional nonsynonymous variants outside the matched core definitions, as well as unmatched coding combinations, were treated conservatively as unresolved. Alleles assigned uncertain or unknown function by CPIC, or carrying unresolved coding combinations, resulted in an indeterminate inferred metabolizer phenotype. These assignments indicate compatibility with observed coding-core definitions rather than definitive identification of complete PharmVar suballeles.

Two sensitivity analyses were performed by refitting the population-adjusted binary logistic regression model of inferred poor versus intermediate or rapid metabolizer status. The first excluded four AMR individuals with indeterminate expanded functional assignments, whereas the second excluded all seven AMR carriers of additional nonsynonymous variants. Native American genetic ancestry was modeled per 10-percentage-point increase, and AMR population was included as a categorical covariate. Python (NumPy and SciPy) was used to maximize the logistic likelihood and calculate Wald confidence intervals and *p*-values. The original population-adjusted association was independently reproduced before applying the sensitivity exclusions.

Extreme-quartile sensitivity comparisons retained the ancestry thresholds defined in the original 347-participant AMR analysis. These procedures were intended to evaluate the robustness of the primary association to additional coding variation observed in the regional dataset, without implying complete variant coverage, definitive functional classification, or experimental validation. The expanded coding-core assignments and both sensitivity analyses are documented in Supplementary Tables S1–S3 (Supplementary File S3). Supplementary File S4 provides an independent Python implementation reproducing the original population-adjusted regression and the four-case exclusion sensitivity analysis using the seven-SNP genotype and ancestry inputs supplied in Supplementary File S1.

## 5. Conclusions

In the four Admixed American populations sampled by the 1000 Genomes Project, higher Native American genetic ancestry was associated with variation in NAT2 seven-SNP haplotypes and lower odds of an inferred poor metabolizer phenotype after adjustment for sampling population. This association persisted after conservative exclusion of individuals with indeterminate expanded coding-variant assignments, supporting its robustness to the additional coding variation observed in the regional dataset. Comparisons across 1000 Genomes macro-populations and linkage disequilibrium analyses provided population-genetic context for these findings. However, the observed associations do not establish causality or predict individual drug responses. The seven-SNP panel also does not permit definitive identification of complete NAT2 suballeles or direct determination of metabolic activity. Further studies involving larger and more diverse populations, comprehensive NAT2 genotyping, local ancestry analysis, and clinical outcomes are needed to clarify the pharmacogenetic implications of these findings. Individual clinical interpretation should rely on NAT2 genotyping and appropriate functional assignments rather than global genetic ancestry estimates.

## Figures and Tables

**Figure 1:**
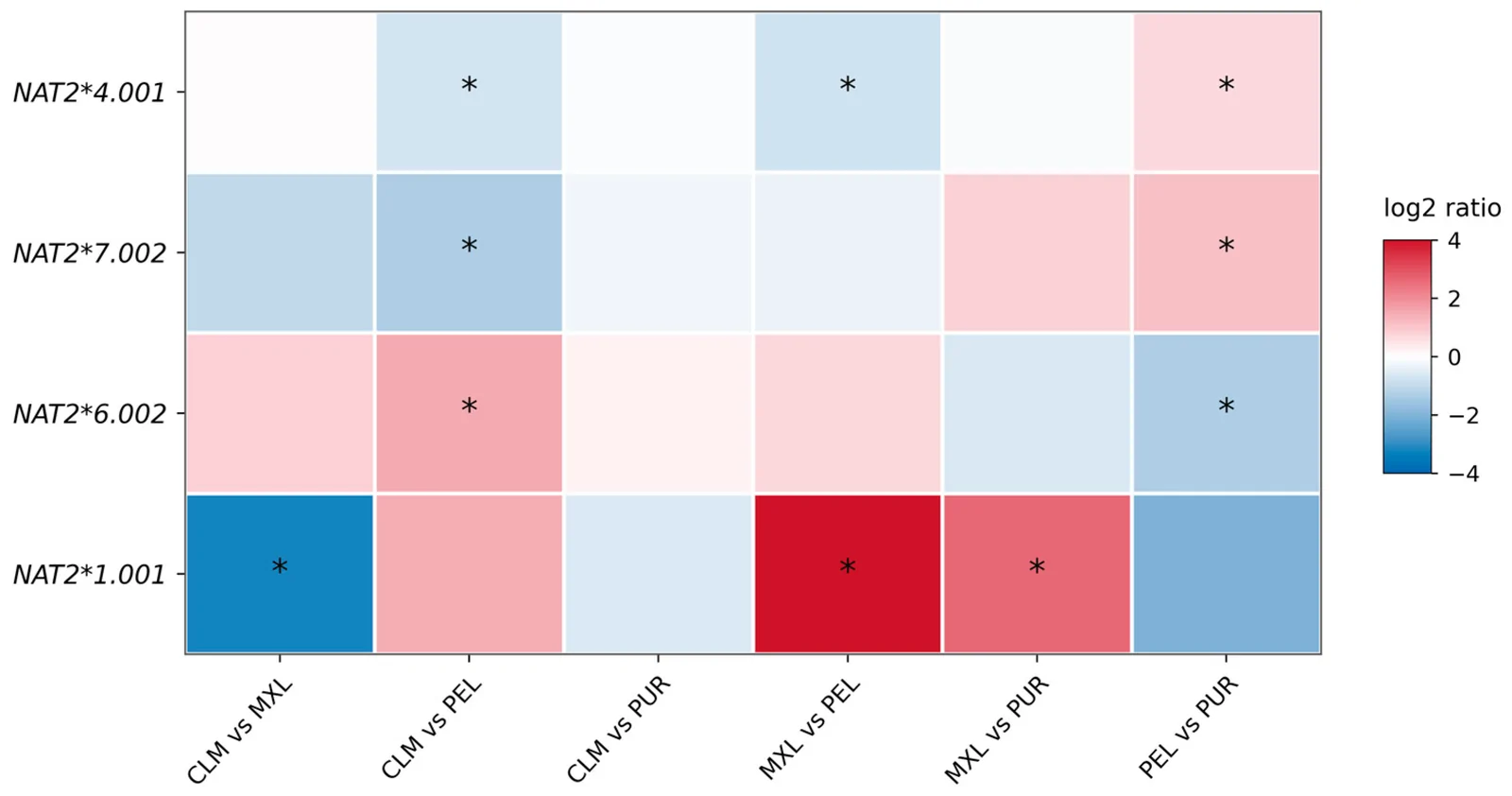
Pairwise *NAT2* haplotype differentiation among Admixed American populations from the 1000 Genomes Project. Heatmap cells represent the log_2_ haplotype frequency ratio for each pairwise comparison among CLM, MXL, PEL, and PUR. Positive values indicate enrichment in the first population named in each comparison, whereas negative values indicate enrichment in the second. Only haplotypes showing at least one significant pairwise difference are displayed. Asterisks indicate comparisons that remained significant after Benjamini–Hochberg correction applied globally across all AMR pairwise tests (FDR < 0.05). Haplotype labels correspond to PharmVar-compatible *NAT2* assignments inferred from the seven-SNP panel.

**Figure 2:**
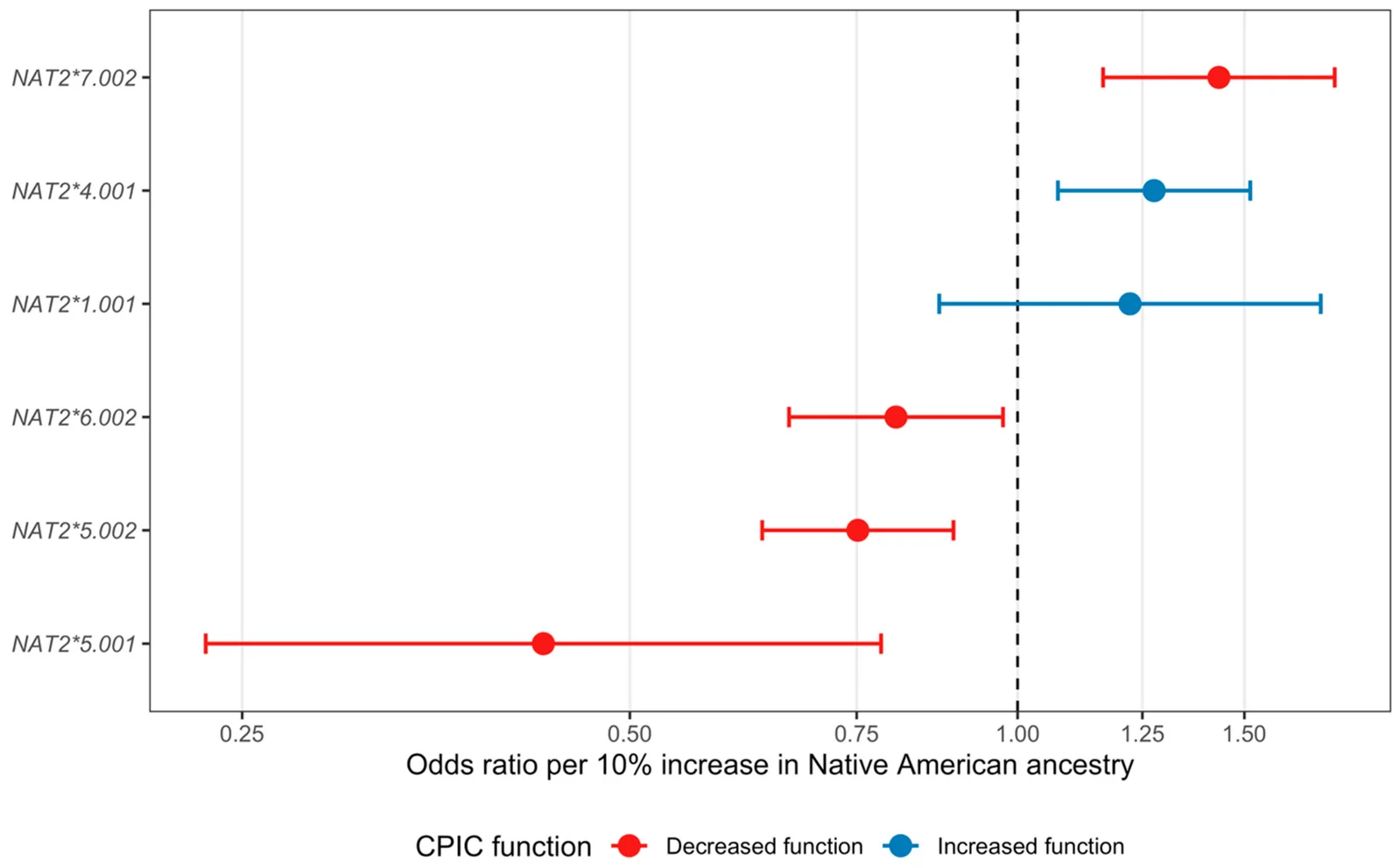
Association between Native American ancestry and major *NAT2* 7-SNP haplotypes within Admixed American populations. Forest plot showing odds ratios and 95% confidence intervals for carriage of each major *NAT2* haplotype per 10-percentage-point increase in Native American ancestry. Estimates were obtained from logistic regression models adjusted for AMR population (CLM, MXL, PEL, and PUR). Odds ratios greater than 1 indicate increasing haplotype carriage with higher Native American ancestry, whereas values below 1 indicate decreasing carriage. Points are colored according to CPIC functional classification of the corresponding PharmVar-compatible *NAT2* assignment. *p*-values were adjusted across haplotypes using the Benjamini–Hochberg method.

**Figure 3:**
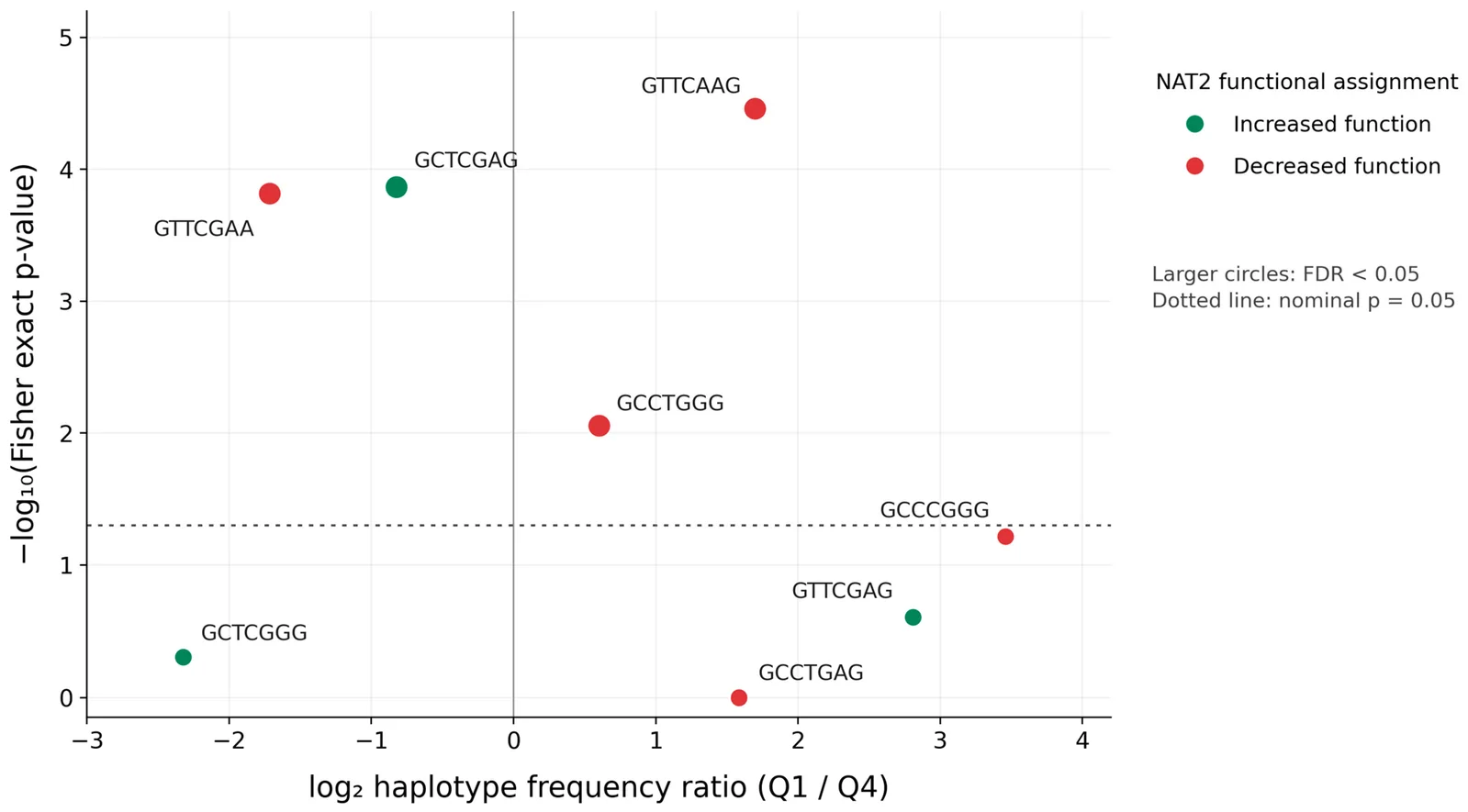
NAT2 haplotype differentiation between extreme Native American ancestry quartiles within Admixed American populations. Volcano plot showing the log_2_ haplotype frequency ratio between the lowest (Q1) and highest (Q4) Native American ancestry quartiles on the x-axis and −log_10_(*p*-value) from Fisher’s exact tests on the y-axis. Positive values indicate enrichment in Q1, corresponding to lower Native American ancestry, whereas negative values indicate enrichment in Q4, corresponding to higher Native American ancestry. Point color indicates the functional classification of the corresponding NAT2 allele. The horizontal dotted line marks *p* = 0.05. Statistical significance reported in the text is based on Benjamini–Hochberg-adjusted FDR values.

**Table 1:** Distribution of major *NAT2* 7-SNP haplotypes among Admixed American populations from the 1000 Genomes Project.

7-SNP Motif	PharmVar-Compatible Allele	Legacy Label	CPIC Function	AMR n (%)	CLM n (%)	MXL n (%)	PEL n (%)	PUR n (%)	χ^2^	FDR
GCCTGGG	NAT2*5.002-compatible	NAT2*5B	Decreased	235 (33.9)	70 (37.2)	47 (36.7)	45 (26.5)	73 (35.1)	5.71	0.253
GCTCGAG	NAT2*4.001-compatible	NAT2*4	Increased	228 (32.9)	53 (28.2)	35 (27.3)	79 (46.5)	61 (29.3)	19.08	0.0010
GTTCAAG	NAT2*6.002-compatible	NAT2*6A	Decreased	119 (17.1)	45 (23.9)	17 (13.3)	14 (8.2)	43 (20.7)	18.77	0.0010
GTTCGAA	NAT2*7.002-compatible	NAT2*7B	Decreased	78 (11.2)	13 (6.9)	18 (14.1)	30 (17.6)	17 (8.2)	13.50	0.0092
GCCCGGG	NAT2*5.001-compatible	NAT2*5C	Decreased	12 (1.7)	3 (1.6)	1 (0.8)	2 (1.2)	6 (2.9)	2.64	0.501
GCTCGGG	NAT2*1.001-compatible	NAT2*12A	Increased	12 (1.7)	1 (0.5)	9 (7.0)	0 (0.0)	2 (1.0)	26.48	7.58 × 10^−5^

Note. Frequencies were calculated at the chromosome level (AMR, n = 694; CLM, n = 188; MXL, n = 128; PEL, n = 170; PUR, n = 208). The table displays the six haplotype motifs with AMR frequencies ≥ 1%. Four additional low-frequency motifs were included in the haplotype-specific testing procedure and grouped as “Other” for the omnibus comparison. χ^2^ values represent Pearson chi-square statistics for haplotype-specific comparisons across the four populations. FDR values were obtained using the Benjamini–Hochberg procedure across the ten observed AMR haplotype motifs. PharmVar-compatible assignments indicate compatibility with the observed seven-SNP motif rather than definitive suballele identification.

**Table 2:** Multiallelic Hardy–Weinberg equilibrium analysis of reconstructed NAT2 diplotypes in the four Admixed American populations.

Population	N	Permutation *p*-Value	FDR	Hₒ	Hₑ
CLM	94	0.142	0.190	0.638	0.719
MXL	64	0.278	0.278	0.750	0.748
PEL	85	0.0070	0.028	0.682	0.676
PUR	104	0.076	0.152	0.644	0.740

Note. Population-specific *p*-values were obtained using 100,000 conditional genotype permutations. FDR values were calculated using the Benjamini–Hochberg procedure across the four populations. Hₒ, observed heterozygosity; Hₑ, expected heterozygosity calculated from population-specific haplotype frequencies. The diplotype-count diagnostics are descriptive; inferential HWE results are based on the conditional permutation test.

**Table 3:** Inferred NAT2 metabolizer phenotype distribution across Native American ancestry quartiles.

Inferred Metabolizer Phenotype	Q1, n (Frequency)	Q4, n (Frequency)
Poor	50 (0.575)	23 (0.264)
Intermediate	26 (0.299)	46 (0.529)
Rapid	11 (0.126)	18 (0.207)

Note. Q1 and Q4 correspond to the lowest and highest Native American ancestry quartiles, respectively. Differences in phenotype distribution were evaluated using a chi-square test of independence (χ^2^ = 17.23, df = 2, *p* = 1.81 × 10^−4^).

**Table 4:** Distribution of major *NAT2* 7-SNP haplotypes across 1000 Genomes macro-populations.

7-SNP Motif	PharmVar-compatible Allele	Legacy Label	CPIC Function	Global n (%)	AFR n (%)	AMR n (%)	EAS n (%)	EUR n (%)	SAS n (%)	χ^2^	FDR
GCTCGAG	NAT2*4.001-compatible	*NAT* *4	Increased	1345 (26.9)	148 (11.2)	228 (32.9)	525 (52.1)	240 (23.9)	204 (20.9)	526.84	1.78 × 10^−111^
GCCTGGG	NAT2*5.002-compatible	NAT2*5B	Decreased	1314 (26.2)	310 (23.4)	235 (33.9)	37 (3.7)	421 (41.8)	311 (31.8)	433.71	1.23 × 10^−91^
GTTCAAG	NAT2*6.002-compatible	NAT2*6A	Decreased	1314 (26.2)	305 (23.1)	119 (17.1)	258 (25.6)	283 (28.1)	349 (35.7)	83.66	7.07 × 10^−17^
GTTCGAA	NAT2*7.002-compatible	NAT2*7B	Decreased	386 (7.7)	38 (2.9)	78 (11.2)	181 (18.0)	22 (2.2)	67 (6.9)	248.53	4.59 × 10^−52^
GTTCGAG	NAT2*4.003-compatible	NAT2*13A	Increased	171 (3.4)	161 (12.2)	4 (0.6)	4 (0.4)	1 (0.1)	1 (0.1)	418.73	1.41 × 10^−88^
GCTCGGG	NAT2*1.001-compatible	NAT2*12A	Increased	163 (3.3)	129 (9.8)	12 (1.7)	2 (0.2)	4 (0.4)	16 (1.6)	246.81	8.99 × 10^−52^
GCCCGGG	NAT2*5.001-compatible	NAT2*5C	Decreased	125 (2.5)	72 (5.4)	12 (1.7)	1 (0.1)	15 (1.5)	25 (2.6)	76.94	1.46 × 10^−15^
ATTCGAG	NAT2*14.002-compatible	NAT2*14B	Decreased	106 (2.1)	104 (7.9)	1 (0.1)	0 (0.0)	1 (0.1)	0 (0.0)	286.72	3.36 × 10^−60^

Note. Only haplotypes with a global frequency ≥1% are shown. Frequencies are calculated at the chromosome level. AFR, African; AMR, Admixed American; EAS, East Asian; EUR, European; SAS, South Asian. PharmVar-compatible assignments indicate the allele/suballele compatible with the observed seven-SNP motif; additional variants not included in the seven-SNP panel may further refine the suballele designation. Statistical heterogeneity across macro-populations was assessed using χ^2^ tests, with Benjamini–Hochberg false-discovery rate correction.

## Data Availability

The Phase 3 genomic data underlying this study are publicly available from the 1000 Genomes Project. Individual genetic ancestry estimates were generated in previously published work as described in Section 4.2. Supplementary File S1 provides the study-specific seven-SNP phased NAT2 VCF, matched individual ancestry and population data, the primary-analysis R script, SHA256 checksums, and execution instructions. Supplementary File S3 provides expanded coding-core compatibility and sensitivity results; Supplementary File S4 provides reproducible Python code for the original and four-case exclusion regressions using the S1 inputs. Regional variants were assessed using the publicly available Phase 3 sequence data and PharmVar/CPIC functional references as described in Section 4.10.

## Supplementary Materials

The following supporting information can be downloaded at: https://www.mdpi.com/article/10.3390/ijms27219884/s1.

## Abbreviations

The following abbreviations are used in this manuscript:

1KGP	1000 Genomes Project
AFR	African
AMR	Admixed American
EAS	East Asian
EUR	European
SAS	South Asian
CLM	Colombians from Medellín
MXL	Mexican Ancestry from Los Angeles
PEL	Peruvians from Lima
PUR	Puerto Ricans from Puerto Rico
*NAT2*	N-acetyltransferase 2
SNP	Single-nucleotide polymorphism
HWE	Hardy–Weinberg equilibrium
LD	Linkage disequilibrium
OR	Odds ratio
CI	Confidence interval
FDR	False-discovery rate
CPIC	Clinical Pharmacogenetics Implementation Consortium
PharmVar	Pharmacogene Variation Consortium
