# Supplementary material for: Native American Ancestry Is Associated with NAT2 Haplotype and Inferred Metabolizer Phenotype Variation in Latin American Populations
Source: Int J Mol Sci. 2026 Nov 4;27(21):9884. doi: 10.3390/ijms27219884 (PMC13763580; doi:10.3390/ijms27219884)
Supplement: Supplementary file 1 [file ijms-27-09884-s001.zip › IJMS_NAT2_S2_Figures.pdf]

Supplementary File S2 — Additional Figures

Native American Ancestry Is Associated with NAT2 Haplotype and Inferred Metabolizer Phenotype Variation in Latin American Populations

Supplementary Figure S1 corresponds to Figure 1 in the original submission. Supplementary Figure S2 corresponds to Figure 5 in the original submission. Both figures are reproduced from the original manuscript; no new analyses are introduced.

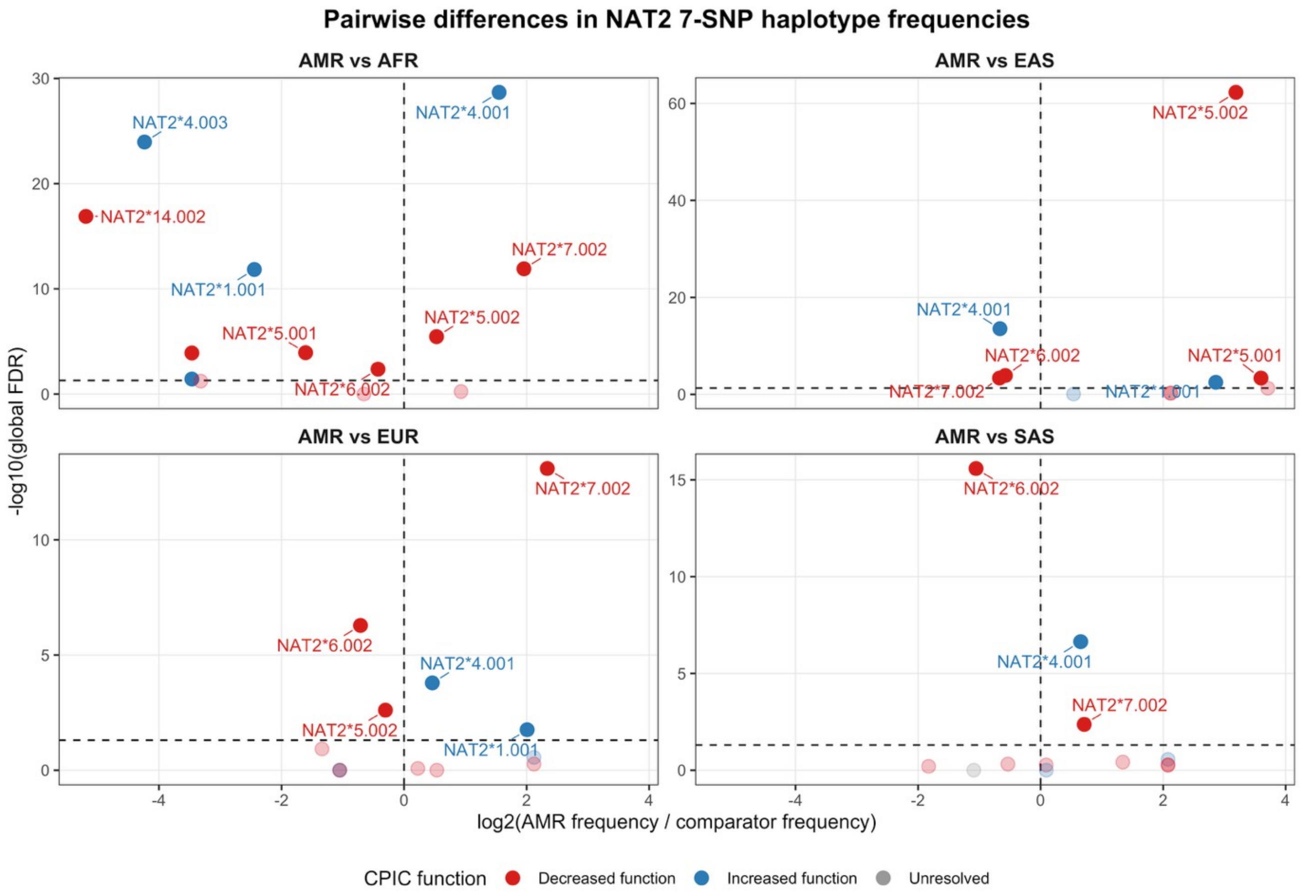

Supplementary Figure S1. Pairwise differences in NAT2 7-SNP haplotype frequencies between Admixed American and other 1000 Genomes macro-populations. Volcano plots compare Admixed American (AMR) individuals with African (AFR), East Asian (EAS), European (EUR), and South Asian (SAS) macro-populations. The x-axis shows the $\log_2$ ratio of haplotype frequencies in AMR relative to the comparator population, with positive values indicating enrichment in AMR and negative values indicating enrichment in the comparator group. The y-axis represents $-\log_{10}$ of the Benjamini–Hochberg false-discovery rate calculated across all 68 pairwise tests. The horizontal dashed line marks FDR = 0.05, and the vertical dashed line indicates no frequency difference. Points are colored according to CPIC functional classification of the corresponding PharmVar-compatible NAT2 allele assignment: increased function, decreased function, or unresolved. Labels are shown for globally significant haplotypes with a global frequency ≥1%.

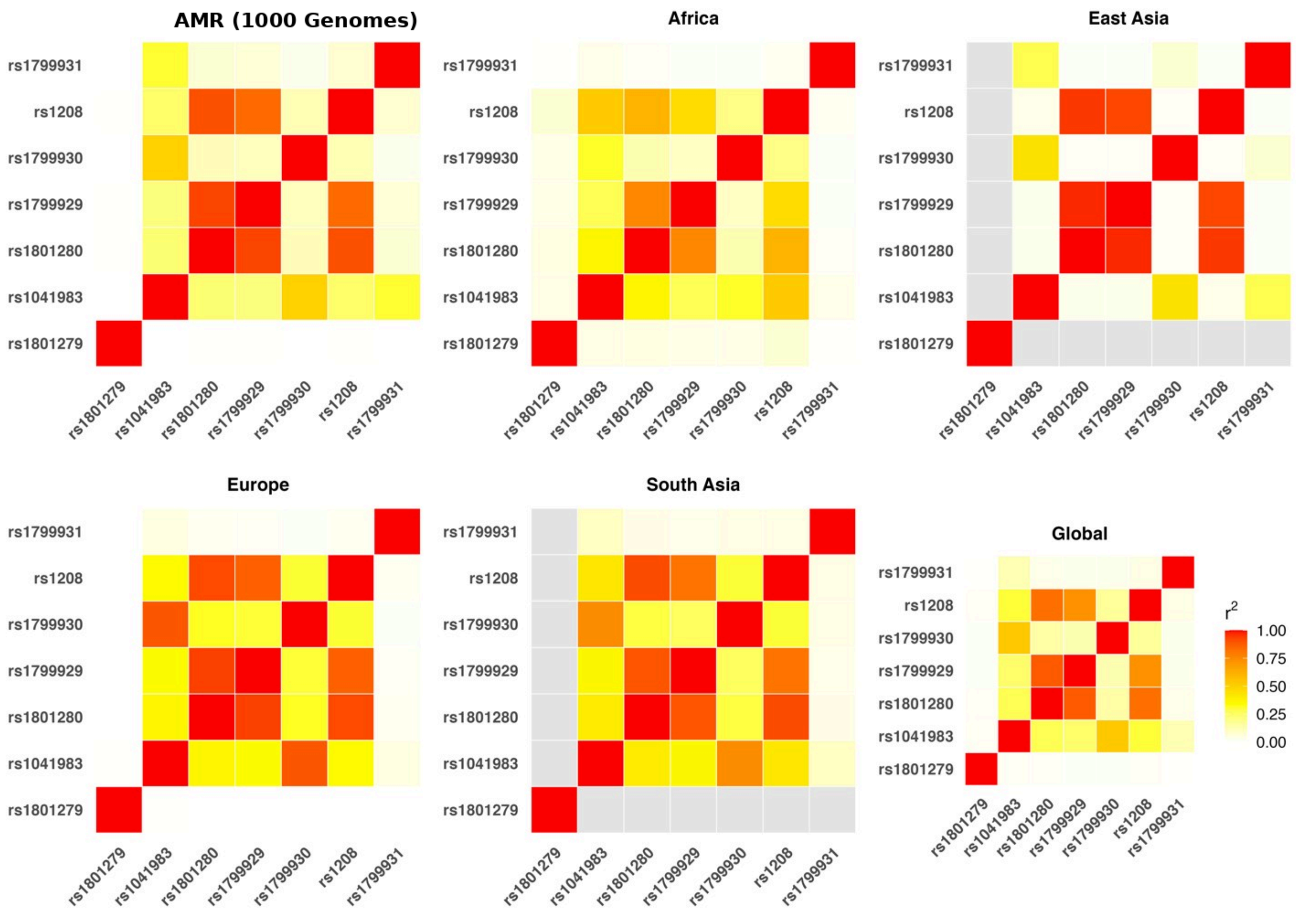

Supplementary Figure S2. Linkage disequilibrium patterns across the NAT2 locus in AMR and global populations. Pairwise linkage disequilibrium (r^2) was calculated among the seven NAT2 SNPs analyzed in this study (rs1801279, rs1041983, rs1801280, rs1799929, rs1799930, rs1208, and rs1799931) across Admixed Americans (AMR), Africans (AFR), East Asians (EAS), Europeans (EUR), South Asians (SAS), and the global 1000 Genomes dataset. Heatmaps display pairwise r^2 values, with increasing color intensity indicating stronger LD. The AMR panel is shown alongside the continental groups to contextualize population differences in LD structure.
