# Supplementary material for: Native American Ancestry Is Associated with NAT2 Haplotype and Inferred Metabolizer Phenotype Variation in Latin American Populations
Source: Int J Mol Sci. 2026 Nov 4;27(21):9884. doi: 10.3390/ijms27219884 (PMC13763580; doi:10.3390/ijms27219884)
Supplement: Supplementary file 1 [file ijms-27-09884-s001.zip › IJMS_NAT2_S3_Tables.pdf]

Supplementary File S3 — Supplementary Tables

Native American Ancestry Is Associated with NAT2 Haplotype and Inferred Metabolizer Phenotype Variation in Latin American Populations

Table S1. Observed expanded coding-core-compatible assignments within each original seven-SNP motif.

Seven-SNP motif	Original core family	Global copies	AMR copies	Expanded compatible core (global copies)
ACTCGAG	NAT2*14	32	1	*14 (32)
ATTCGAG	NAT2*14	106	1	*14 (96); *47 (9); Unresolved (1)
ATTCGGG	NAT2*46	1	0	*46 (1)
GCCCGGG	NAT2*5	125	12	*33 (2); *5 (123)
GCCTAGG	Unresolved	1	0	Unresolved (1)
GCCTGAG	NAT2*16	26	4	*16 (26)
GCCTGGG	NAT2*5	1314	235	*31 (3); *33 (2); *49 (4); *5 (1303); Unresolved (2)
GCTCAAG	NAT2*6	10	0	*36 (9); *6 (1)
GCTCGAA	NAT2*7	1	0	*7 (1)
GCTCGAG	NAT2*4	1345	228	*10 (1); *4 (1338); *55 (1); *57 (1); *60 (1); Unresolved (3)
GCTCGGG	NAT2*1	163	12	*1 (124); *43 (14); *44 (23); *45 (1); *51 (1)
GCTTGGG	NAT2*1	1	0	*1 (1)
GTTCAAG	NAT2*6	1314	119	*37 (3); *38 (8); *39 (46); *53 (1); *56 (1); *58 (1); *6 (1252); *63 (1); *64 (1)
GTTCAGG	NAT2*34	2	0	*34 (2)
GTTCGAA	NAT2*7	386	78	*59 (1); *7 (384); Unresolved (1)
GTTCGAG	NAT2*4	171	4	*4 (170); *61 (1)
GTTCGGG	NAT2*1	10	0	*1 (5); *41 (5)

Counts refer to chromosome copies (global total = 5,008; AMR total = 694). The motif order is rs1801279, rs1041983, rs1801280, rs1799929, rs1799930, rs1208, and rs1799931. Asterisk labels denote NAT2 coding-core compatibility, not definitive full suballeles. Unresolved includes unmatched core combinations or additional amino-acid-changing variants not covered by a matched definition. Functional classification further identifies uncertain or unknown function among otherwise compatible cores. Across the cohort, 57 chromosome copies in 56 individuals had uncertain (45 copies), unknown (4), or unresolved (8) assignments.

Table S2. AMR individuals with indeterminate functional assignments after expanded coding-variant assessment.

Sample / population	Original phenotype	Affected coding haplotype	Expanded interpretation
HG01187 / PUR	Rapid	c.403C>G	NAT2*44-compatible; uncertain function
NA19761 / MXL	Intermediate	c.609G>T	NAT2*43-compatible; uncertain function
HG01556 / CLM	Intermediate	c.566T>C; c.803G>A	Unresolved coding combination
HG01917 / PEL	Intermediate	c.29T>C; c.282C>T; c.803G>A; c.857G>A	Unresolved coding combination

Each affected haplotype is shown relative to the reference coding sequence. The two unresolved combinations are not proposed as new star alleles. Expanded assessment excluded these four participants from the conservative phenotype sensitivity analysis; the primary seven-SNP analysis retains its original calls.

Table S3. Sensitivity of the AMR ancestry association to additional observed coding variation.

Analysis	n	OR	95% CI	p-value
Original seven-SNP analysis	347	0.785	0.660–0.933	0.0060
Exclude four indeterminate expanded assignments	343	0.796	0.669–0.946	0.0097
Exclude all seven additional nonsynonymous-variant carriers	340	0.796	0.670–0.947	0.0100

Binary logistic regression: inferred poor versus intermediate/rapid metabolizer status, adjusted for AMR population. Odds ratios are per 10-percentage-point increase in Native American ancestry. Confidence intervals and p-values are Wald estimates. The additional three excluded carriers in the final row retained decreased-function assignments under the expanded assessment. These analyses do not establish clinical validity of the seven-SNP panel.
